# Rottlerin inhibits cell growth and invasion via down-regulation of Cdc20 in glioma cells

**DOI:** 10.18632/oncotarget.11974

**Published:** 2016-09-12

**Authors:** Lixia Wang, Yingying Hou, Xuyuan Yin, Jingna Su, Zhe Zhao, Xiantao Ye, Xiuxia Zhou, Li Zhou, Zhiwei Wang

**Affiliations:** ^1^ The Cyrus Tang Hematology Center and Collaborative Innovation Center of Hematology, Jiangsu Institute of Hematology, The First Affiliated Hospital, Soochow University, Suzhou, China; ^2^ Department of Gynecologic Oncosurgery, Jilin Province Cancer Hospital, Changchun, Jilin, China; ^3^ Department of Pathology, Beth Israel Deaconess Medical Center, Harvard Medical School, USA

**Keywords:** rottlerin, Cdc20, glioma, growth, invasion

## Abstract

Rottlerin, isolated from a medicinal plant *Mallotus phillippinensis*, has been demonstrated to inhibit cellular growth and induce cytoxicity in glioblastoma cell lines through inhibition of calmodulin-dependent protein kinase III. Emerging evidence suggests that rottlerin exerts its antitumor activity as a protein kinase C inhibitor. Although further studies revealed that rottlerin regulated multiple signaling pathways to suppress tumor cell growth, the exact molecular insight on rottlerin-mediated tumor inhibition is not fully elucidated. In the current study, we determine the function of rottlerin on glioma cell growth, apoptosis, cell cycle, migration and invasion. We found that rottlerin inhibited cell growth, migration, invasion, but induced apoptosis and cell cycle arrest. Mechanistically, the expression of Cdc20 oncoprotein was measured by the RT-PCR and Western blot analysis in glioma cells treated with rottlerin. We observed that rottlerin significantly inhibited the expression of Cdc20 in glioma cells, implying that Cdc20 could be a novel target of rottlerin. In line with this, over-expression of Cdc20 decreased rottlerin-induced cell growth inhibition and apoptosis, whereas down-regulation of Cdc20 by its shRNA promotes rottlerin-induced anti-tumor activity. Our findings indicted that rottlerin could exert its tumor suppressive function by inhibiting Cdc20 pathway which is constitutively active in glioma cells. Therefore, down-regulation of Cdc20 by rottlerin could be a promising therapeutic strategy for the treatment of glioma.

## INTRODUCTION

Cerebral glioma is the most common brain tumor, which is one of the top ten malignant tumors in human [1]. Among primary malignant brain tumors, 80 percent tumors are malignant gliomas (MGs). Glioblastoma multiforme (GBM) accounts for more than half of MG cases [2]. MGs have been reported to be associated with high morbidity and mortality [3]. Multiple studies have demonstrated that many potential risk factors could contribute to brain tumors, although detailed explanation how these factors are involved in brain tumorigenesis is unclear. The most important factors of brain tumor development include exposure to ionizing radiation, and history of allergy or atopic disease [4]. Surgery is often difficult to remove entire brain tumor and chemotherapeutic drugs cannot pass the blood-brain barrier [5]. Therefore, current combinations of surgical resection, radiation therapy and chemotherapy regimens fail to significantly improve long-term patient survival for these brain cancers [6]. Thus, it is pivotal to discover the new therapeutic agents for the treatment of glioma.

Rottlerin, also called mallotoxin, is isolated from plant *Mallotus phillippinensis* [7]. Initial studies identified that rottlerin is an inhibitor of PKCδ (protein kinase C δ) [8]. In recent years, increasing evidence implicated that rottlerin exhibited its anti-tumor activity through inhibition of cell proliferation [9] and migration [10], induction of apoptosis [11] and cell cycle arrest [12] in a variety of human cancer cells. Interestingly, rottlerin was discovered to induce autophagy and apoptotic cell death through PKCδ-independent pathway [13]. Li et al. also found that rottlerin induced proapoptotic endoplasmic reticulum stress via PKCδ-independent pathway in human colon cancer cells [14]. Similarly, rottlerin induced apoptosis through upregulation of DR5 (death receptor 5) via PKCδ-independent pathway in human malignant tumor cells [14]. Mechanistically, rottlerin was reported to inhibit the NF-κB (nuclear factor kappa B)/cyclin D1 cascade in breast cancer cells [15]. Moreover, Ohno et al. found that rottlerin stimulates apoptosis in pancreatic cancer cells through disrupting the interactions between prosurvival Bcl-2 proteins and proapoptotic BH3-only proteins [16]. Although multiple studies identified the molecular insight onto rottlerin-induced tumor suppression, the underlying mechanisms are still elusive.

Ubiquitination by the UPS (ubiquitin proteasome system) controls cell cycle progression via protein degradation [17]. APC/C (anaphase promoting complex/C) is a multi-subunit E3 ubiquitin ligase, which forms APC^Cdc20^ to exert its biological functions largely through targeting its downstream substrates for ubiquitination and subsequent degradation [18–20]. Emerging evidence has demonstrated that Cdc20 (cell division cycle 20) has an oncogenic function in tumorigenesis [18]. Overexpression of Cdc20 has been identified in a broad spectrum of human cancers and is associated with poor prognosis in various types of cancers [21–23]. For example, overexpression of Cdc20 was observed in glioblastomas, whereas Cdc20 was under-expressed in low-grade gliomas [24]. Furthermore, Cdc20 level was significantly correlated with glioma grade and survival time [25]. Mechanistically, it has been found that APC/C (Cdc20) controls the ubiquitin-mediated degradation of p21 in prometaphase [26]. In addition, one study reported that Cdc20-mediated degradation of conductin regulated Wnt/beta-catenin signaling for maximal activity during G1/S [27]. Moreover, Cdc20 has been identified to be negatively regulated by p53 [28]. These reports indicated that Cdc20 could be a potential therapeutic target for combating human cancers.

In the current study, we investigated whether Cdc20 plays an important role in regulation of cell growth, apoptosis, cell cycle, migration and invasion in glioma cells. Moreover, we explored whether rottlerin could inhibit the expression of Cdc20 in glioma cells. Furthermore, we determined whether rottlerin exerts its anticancer function via inactivation of Cdc20 in glioma cells. We found that rottlerin suppressed cell growth and induced apoptosis and cell cycle arrest in glioma cell lines. We also demonstrated that rottlerin could down-regulate the expression of Cdc20, leading to anti-tumor activity in glioma cells. Therefore, rottlerin could be a potential efficient agent to inhibit Cdc20 in glioma.

## RESULTS

### Rottlerin inhibited glioma cell proliferation

**Figure 1:**
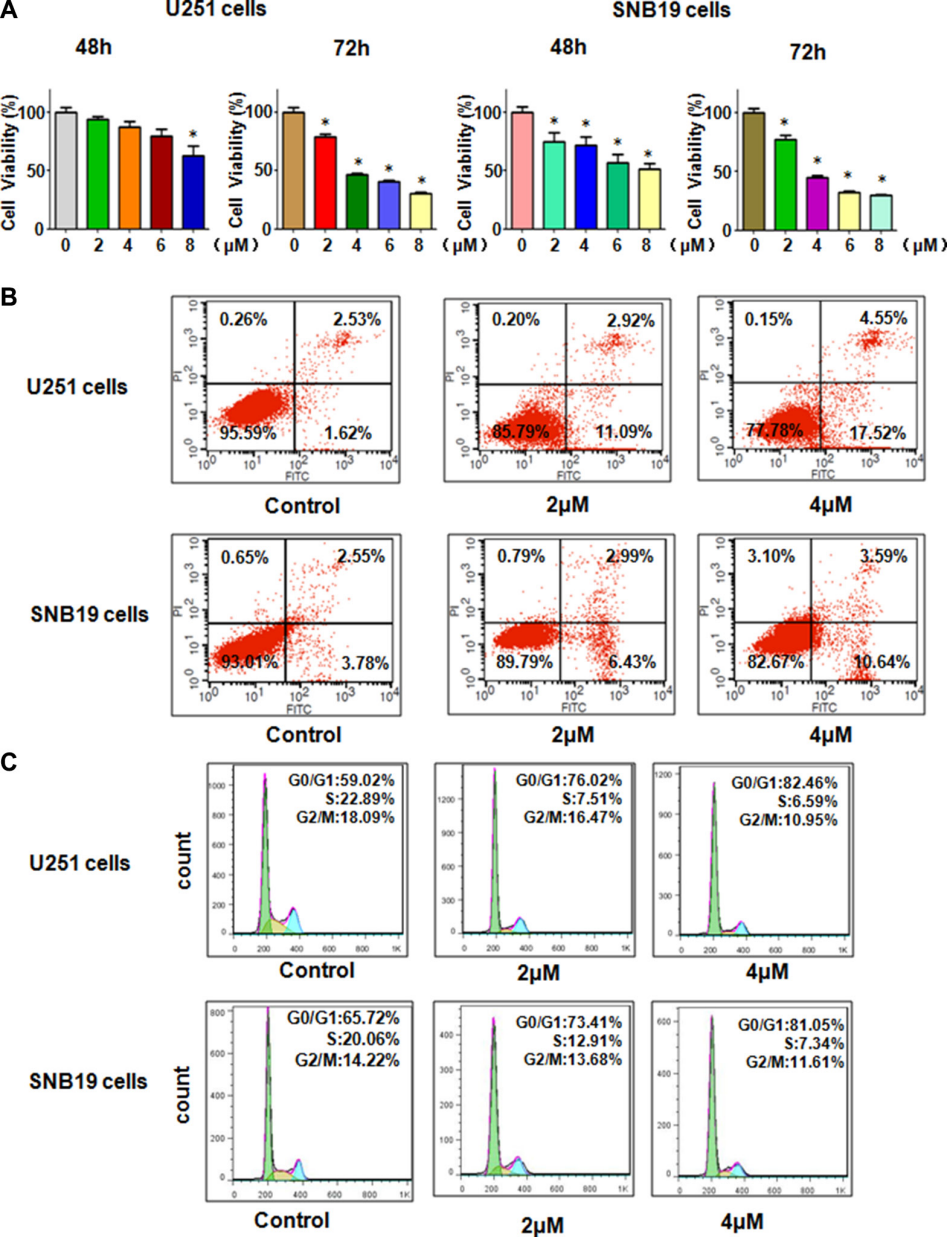
Effect of Rottlerin on cell growth, apoptosis, and cell arrest (**A**) MTT assay was used to detect the cell growth in glioma cells treated with rottlerin. *P < 0.05, compared to the control. (**B**) Cell apoptosis was conducted by FACS in glioma cells treated with rottlerin. (**C**) Cell cycle analysis was determined by Flow cytometry in rottlerin-treated glioma cells.

Rottlerin has been reported to exhibit anti-proliferation in human cancer cells. To determine whether rottlerin could inhibit the glioma cells growth, MTT assay was performed in U251 and SNB19 glioma cells treated with different concentrations of rottlerin for 48 h and 72 h. We observed that rottlerin treatment caused cell growth inhibition in the time- and dose- dependent manners in glioma cells (Figure 1A). Our MTT results have clearly demonstrated that rottlerin inhibited cell proliferation in glioma cells.

### Rottlerin induced cell apoptosis in glima cells

Next, we determine whether rottlerin could trigger cell apoptosis in glioma cells, PI-FITC-annexin assay was conducted in U251 and SNB19 glioma cells treated with 2 μM and 4 μM rottlerin for 48 hours. We found that 2 μM and 4 μM rottlerin induced cell apoptosis from 4.15% to 14.01%, to 22.07%, respectively, in U251 cells (Figure 1B). Similarly, 4 μM rottlerin treatments led to cell apoptosis from 6.33% to 14.23% in SNB19 cells (Figure 1B). These results indicated that rottlerin stimulated cell apoptosis in glioma cells.

### Rottlerin induced cell cycle arrest in glioma cells

To dissect whether rottlerin treatment could arrest cell cycle in glioma cells, cell cycle analysis with PI staining and flow cytometry was used to test the cell cycle in U251 and SNB19 cells after 2 μM and 4 μM rottlerin treatments for 48 h. We found that it has a typical G_0_/G_1_ cycle arrest in the rottlerin-treated glioma cells (Figure 1C). Specifically, 2 μM and 4 μM rottlerin led to G1 cell population from 59% to 77% to 82%, respectively, in U251 cells (Figure 1C). Similarly, 2 μM and 4 μM rottlerin treatments caused G1 cell population from 65.7% to 73.4% to 81%, respectively, in SNB19 cells (Figure 1C). These findings suggest that rottlerin induced G1 cell cycle arrest in glioma cells.

### Rottlerin inhibited cell migration and invasion

**Figure 2:**
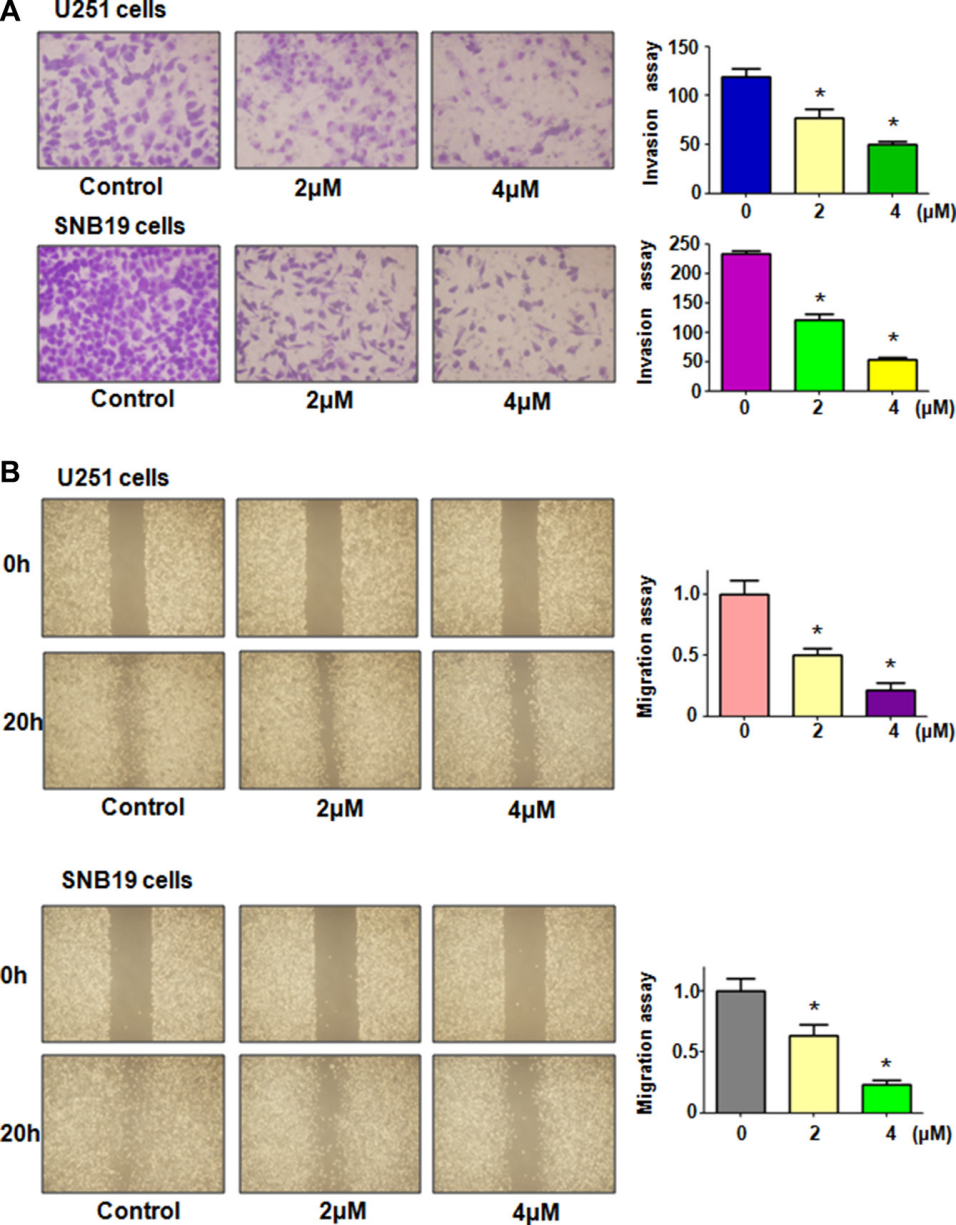
Rottlerin inhibited cell migration and invasion in glioma cells (**A**) Left panel: The inhibitory effect of rottlerin on glioma cell invasion was determined by Transwell chambers assay in U251 cells (Top panel) and SNB19 cells (Bottom panel). Right panel: Quantitative results are illustrated for left panel. (**B**) Left panel: Cell migration was detected using wound healing assay in U251 cells (Top panel) and SNB19 cells (Bottom panel) after rottlerin treatment. Quantitative results are illustrated for left panel. *P < 0.05, **P < 0.01 vs control.

To explore whether rottlerin plays a key role in regulation of motility of glioma cells, invasion assay was performed in glioma cells treated with rottlerin for 20 hours. We found that rottlerin significantly retarded the penetration of glioma cells through the Matrigel-coated membrane in a dose-dependent manner, suggesting that rottlerin inhibited cell invasion in glioma cells (Figure 2A). To further investigate the inhibition of migration in glioma cells by rottlerin, scratch assay was used to detect the migratory activity in glioma cells after rottlerin treatment. The results from our wound healing assay showed that rottlerin remarkably suppressed cell migration in a dose-dependent manner in both glioma cell lines (Figure 2B). Altogether, rottlerin inhibited cell motility in glioma cells.

### Rottlerin decreased Cdc20 expression

**Figure 3:**
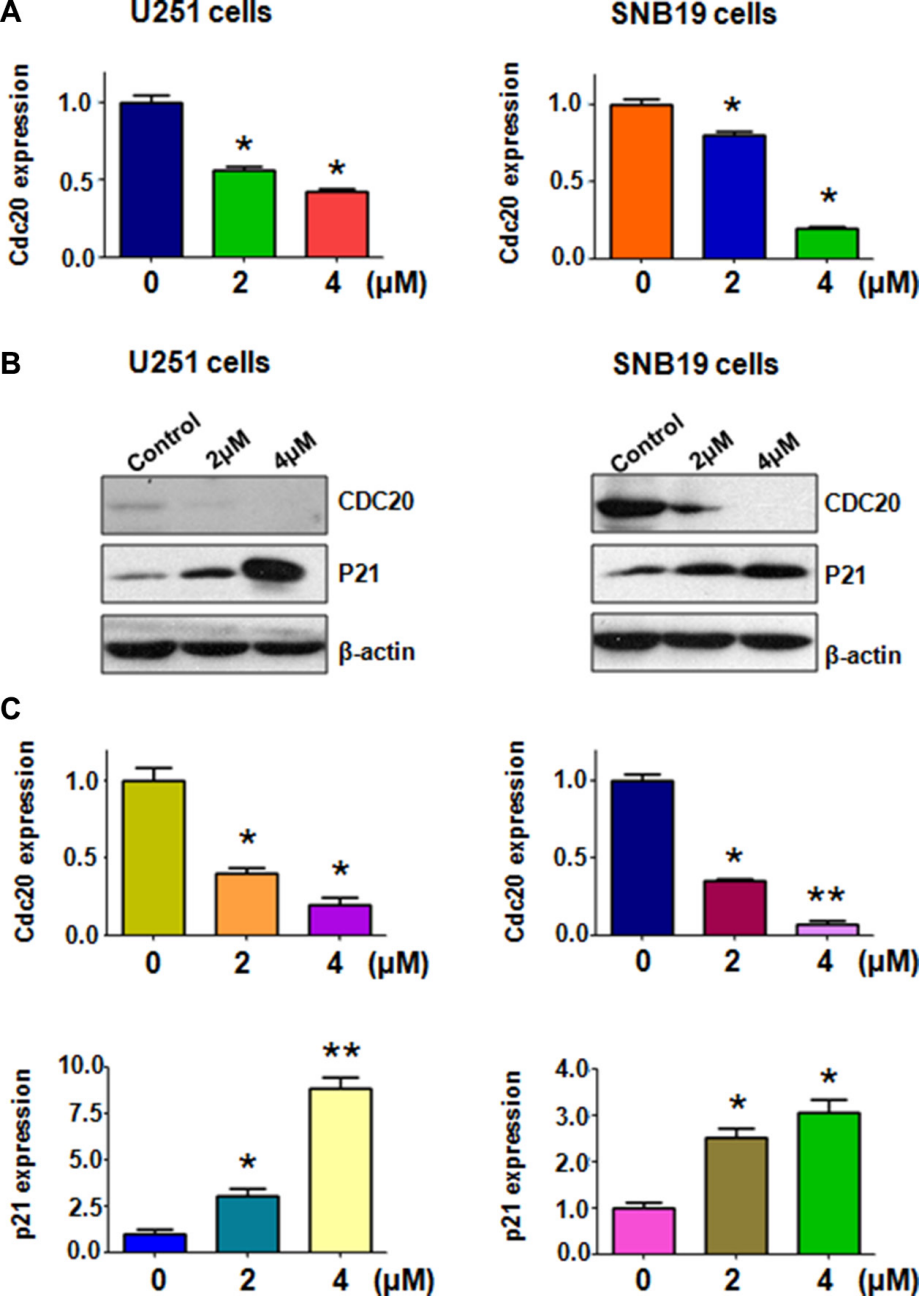
Rottlerin downregulated Cdc20 expression at RNA and protein levels (**A**) The mRNA level of Cdc20 was measured using RT-PCR in glioma cells treated with rottlerin. *P < 0.05, **P < 0.01 vs control. (**B**) The expression of Cdc20 and p21 was determined by Western blotting analysis in U251 and SNB19 cells after rottlerin treatment. (**C)** Quantitative results are illustrated for left panel. *P < 0.05, **P < 0.01, compared to the control.

Emerging evidence has demonstrated that Cdc20 plays an oncogenic role in the development and progression of human cancers [18]. Further studies revealed that targeting Cdc20 could be a novel therapeutic approach in human cancers [29]. Therefore, we explored whether rottlerin could down-regulate the expression of Cdc20 in glioma cells. RT-PCR and Western blotting analysis were used to detect the expression of Cdc20 in glioma cells after rottlerin treatment for 72 hours. Our RT-PCR results showed that Cdc20 mRNA level was down-regulated by rottlerin in U251 and SNB19 cells (Figure 3A). Moreover, our Western blotting analysis revealed that rottlerin significantly decreased Cdc20 protein levels in glioma cells (Figure 3B and 3C). Importantly, we also found that p21, one of Cdc20 targets, was increased in both glioma cells after rottlerin treatment (Figure 3B and 3C). Our findings demonstrated that rottlerin could be a potential inhibitor of Cdc20 in glioma cells.

### Over-expression of Cdc20 reversed the anti-proliferation of rottlerin

**Figure 4:**
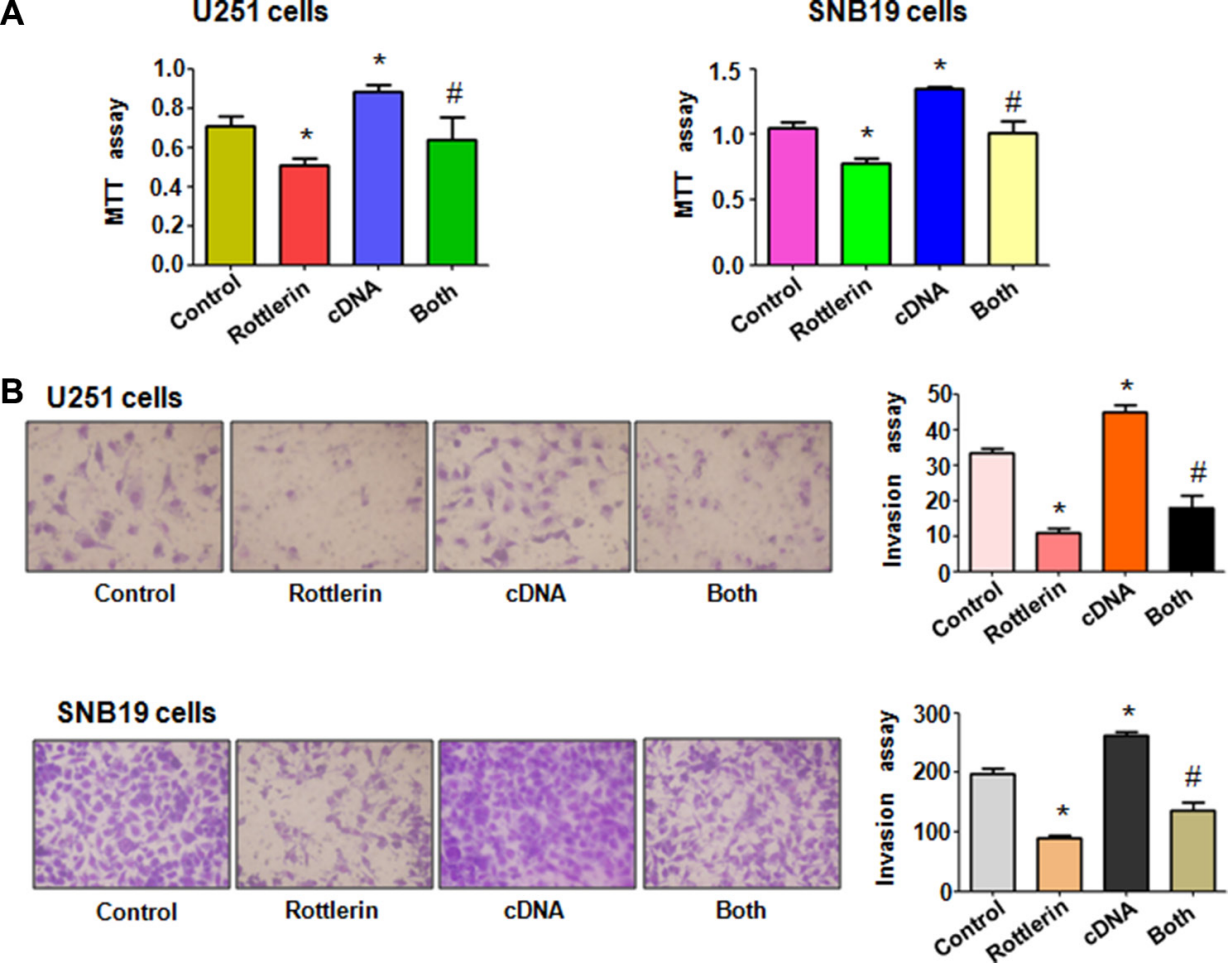
The effect of Cdc20 overexpression on cell growth and invasion (**A**) MTT assay was used to detect the effect of Cdc20 overexpression in combination with rottlerin treatment on glioma cell proliferation. Control: pcDNA 3.1 treatment; Rottlerin: 2 μM rottlerin; cDNA: Cdc20 cDNA; Both: rottlerin + Cdc20 cDNA. *P < 0.05, **P < 0.01, compared with control; ^#^*P* < 0.05 compared with rottlerin treatment or Cdc20 cDNA transfection. (**B**) Left panel, Invasion assay was performed in glioma cells after Cdc20 cDNA transfection and rottlerin treatment. Right panel, Quantitative results are illustrated for left panel.

To validate the function of Cdc20 in rottlerin-mediated cell growth inhibition, Cdc20 cDNA or empty vector as control was transfected into glioma cells followed by rottlerin treatment for 48 hours. We found that overexpression of Cdc20 promoted cell growth in both glioma cells (Figure 4A). More importantly, we observed that Cdc20 overexpression abrogated cell growth inhibition induced by rottlerin in glioma cells (Figure 4A). These findings indicated that rottlerin exerts its anti-proliferation of glioma cells via inhibition of Cdc20 in glioma cells.

### Over-expression of Cdc20 abrogated anti-motility of rottlerin in glioma cells

**Figure 5:**
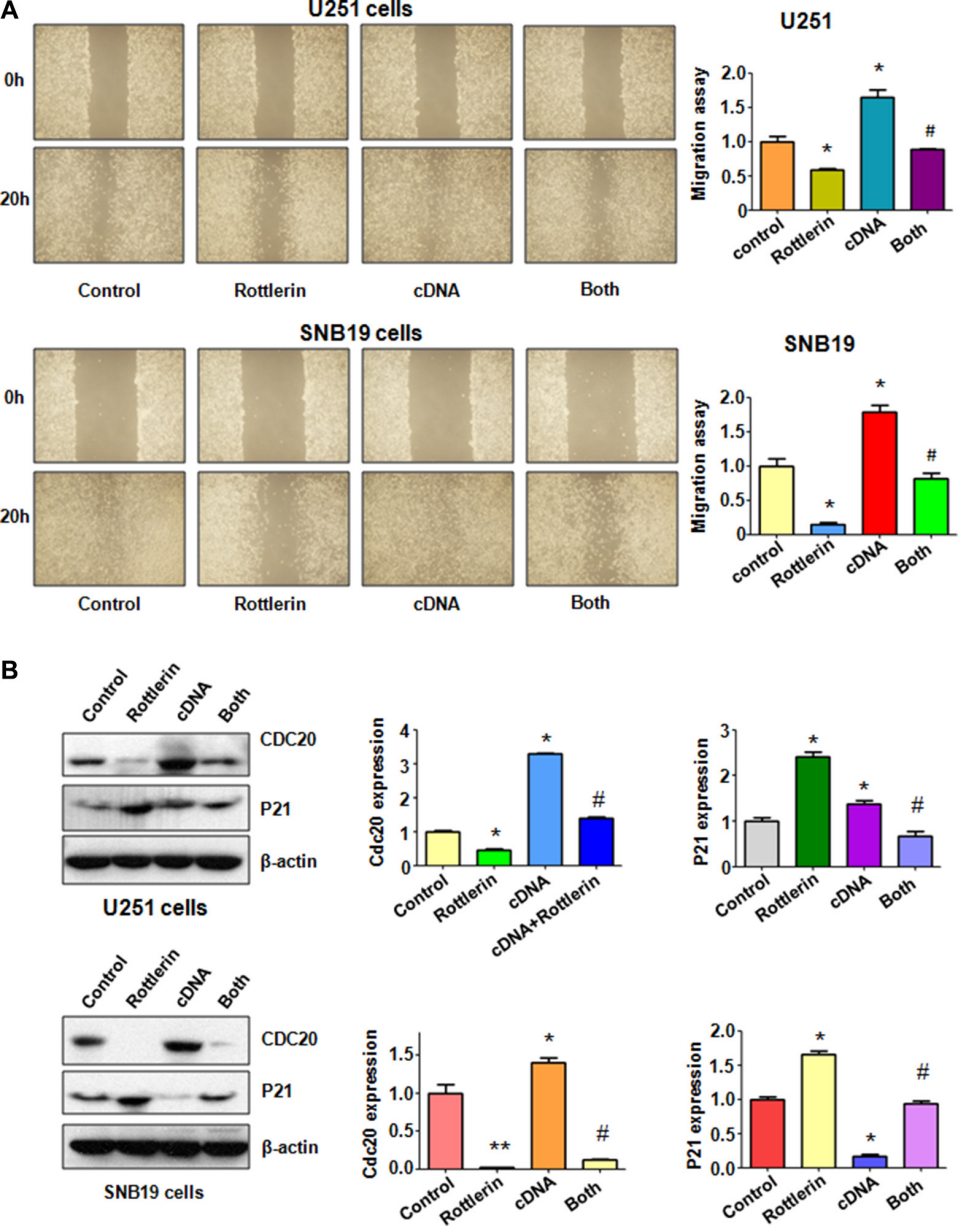
The effect of Cdc20 overexpression on cell migration in glioma cells (**A**) Left panel: The wound healing assay was conducted to detect the cell migration in glioma cells after Cdc20 cDNA transfection and rottleirn treatment. Right panel: Quantitative results are illustrated for left panel. Control: pcDNA 3.1 treatment; Rottlerin: 2 μM rottlerin; cDNA: Cdc20 cDNA; Both: rottlerin + Cdc20 cDNA. *P < 0.05, **P < 0.01, compared with control; ^#^*P* < 0.05 compared with rottlerin treatment or Cdc20 cDNA transfection. (**B**) Left panel: The expression of Cdc20 and its target p21 was detected by western blotting in glioma cells with Cdc20 cDNA transfection and rottlerin treatment. Right panel: Quantitative results are illustrated for left panel.

To measure the effect of Cdc20 overexpression on cell invasion, Matrigel invasion assay was conducted in glioma cells after rottlerin treatment and Cdc20 cDNA transfection. We found that overexpression of Cdc20 significantly enhanced cell invasion in both U251 cells and SNB19 cells (Figure 4B). Moreover, Cdc20 overexpression abrogated inhibition of cell invasion induced by rottlerin in glioma cells (Figure 4B). To further explore whether Cdc20 could control cell motility in glioma cell lines, scratch assay was performed in glioma cells treated with rottlerin in combination with Cdc20 cDNA transfection. Our scratch assay showed that over-expression of Cdc20 promoted the glioma cell migration (Figure 5A). Overexpression of Cdc20 reversed the cell migration inhibition in rottlerin-treated glioma cells (Figure 5A). Importantly, we found that Cdc20 cDNA transfection upregulated Cdc20 expression in glioma cells (Figure 5B). Additionally, Cdc20 overexpression abrogated the inhibition of Cdc20 in glioma cells treated with rottlerin (Figure 5B). Consistently, p21 expression is downregulated in Cdc20-transfected glioma cells (Figure 5B). Similarly, Cdc20 cDNA transfection reversed the upregulation of p21 by rottlerin in both glioma cells (Figure 5B). Our data suggested that rottlerin inhibited cell motility partly through inhibition of Cdc20 in glioma cells.

### Down-regulation of Cdc20 by its shRNA promoted rottlerin-induced antitumor activity

**Figure 6:**
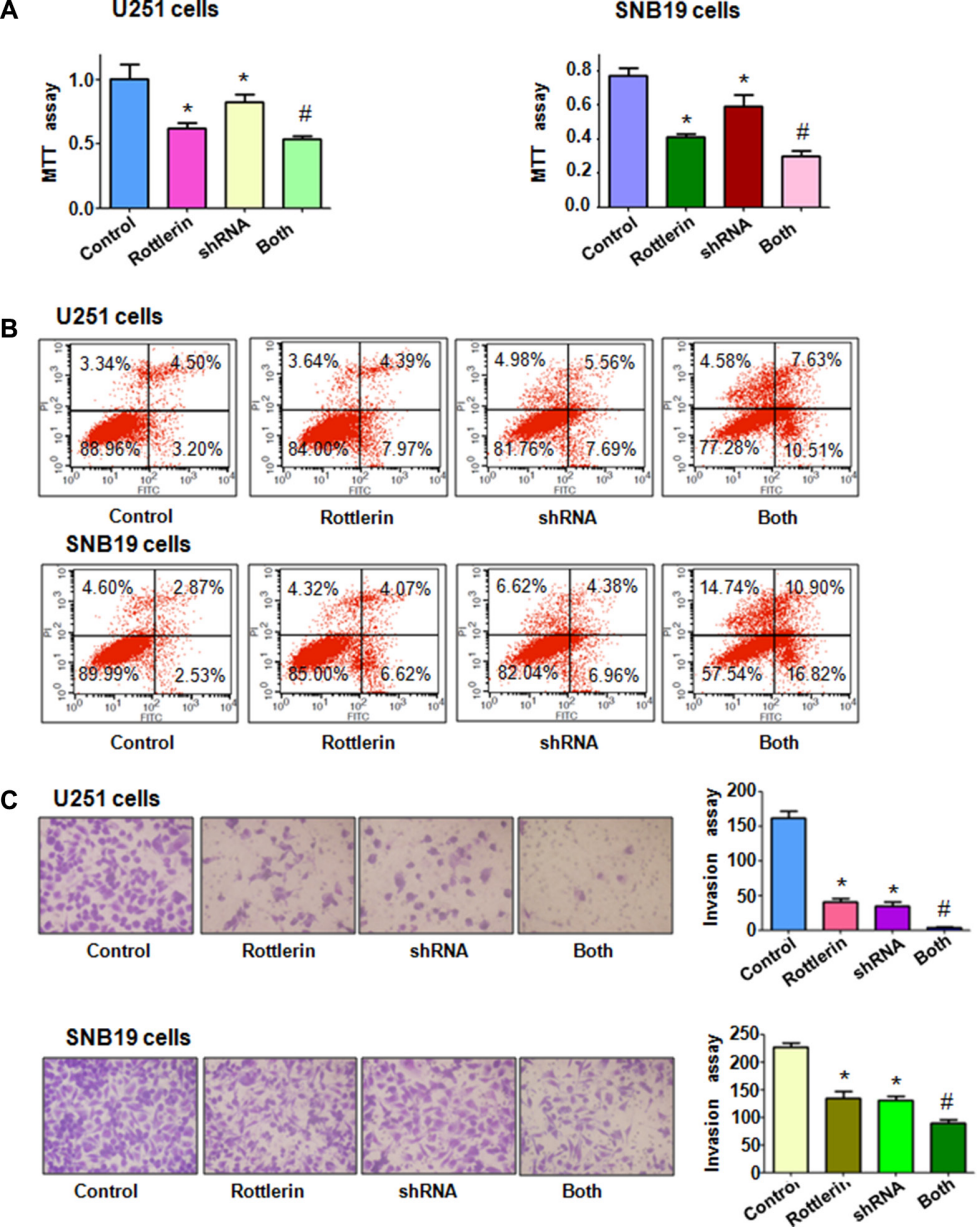
The effect of Cdc20 downregulation on cell growth, apoptosis and invasion (**A**) MTT assay was used to detect the effect of Cdc20 shRNA in combination with rottlerin treatment on glioma cell proliferation. Control: siRNA control; Rottlerin: 2 μM rottlerin; shRNA: Cdc20 shRNA; Both: rottlerin + Cdc20 shRNA.*P < 0.05, compared with control; ^#^*P* < 0.05 compared with rottlerin treatment or Cdc20 shRNA. (**B**) Apoptosis was detected by Flow cytometry in glioma cells with Cdc20 shRNA and rottlerin treatment. (**C**) Invasion assay was performed in glioma cells after Cdc20 shRNA and rottlerin treatment.

**Figure 7:**
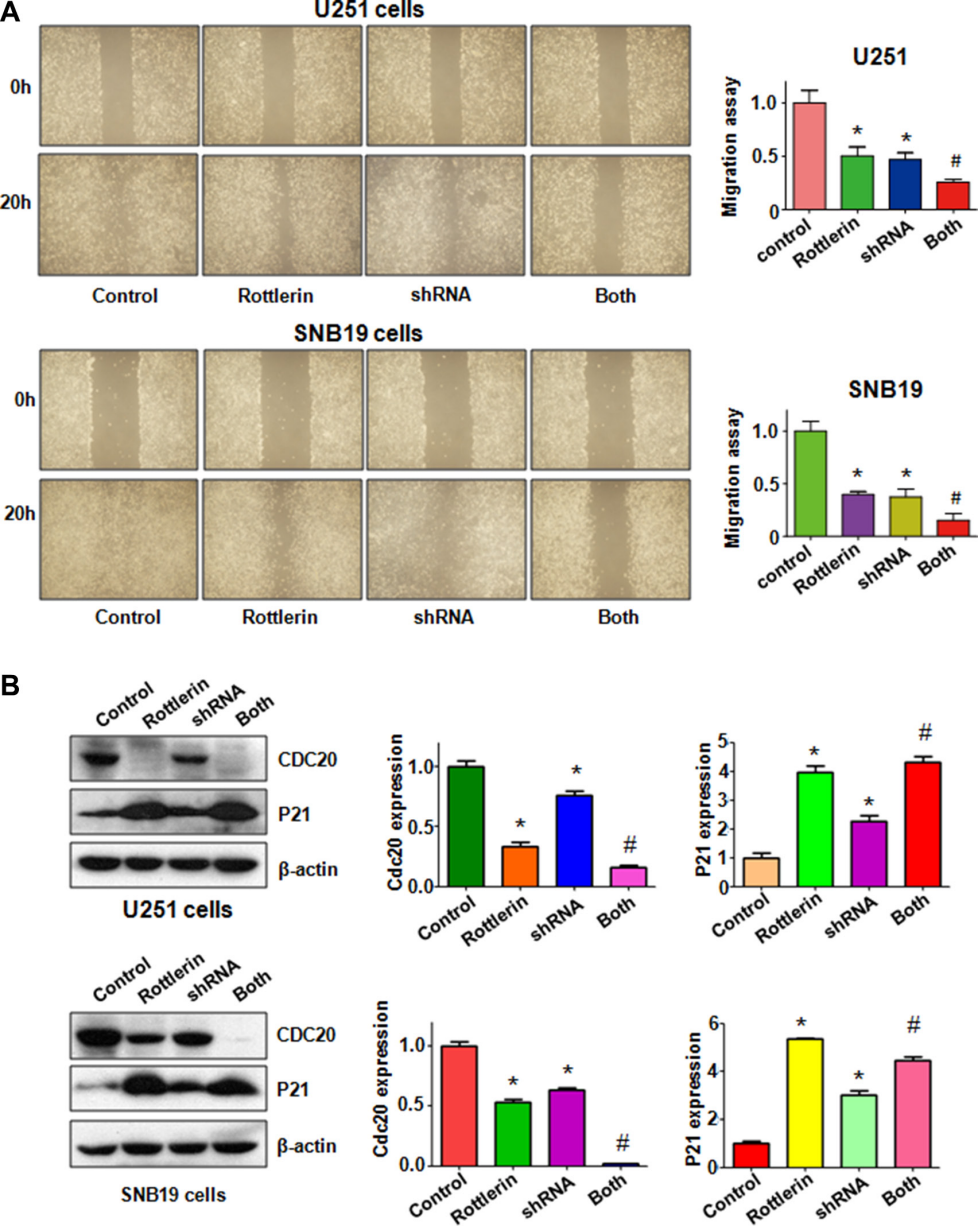
The effect of Cdc20 downregulation on cell migration in glioma cells (**A**) Left panel: The wound healing assay was conducted to detect the cell migration in glioma cells after Cdc20 shRNA and rottlerin treatment. Control: siRNA control; Rottlerin: 2 μM rottlerin; shRNA: Cdc20 shRNA; Both: rottlerin + Cdc20 shRNA. Right panel: Quantitative results are illustrated for left panel. *P < 0.05, vs control; ^#^*P* < 0.05 vs Cdc20 shRNA treatment or rottlerin treatment. (**B**) Left panel: The expression of Cdc20 and its target p21 was detected by western blotting in glioma cells with Cdc20 shRNA and rottlerin treatment. Right panel: Quantitative results are illustrated for left panel. *P < 0.05, **P < 0.01, compared with control; ^#^*P* < 0.05 compared with rottlerin treatment or Cdc20 shRNA treatment.

To further determine the role of Cdc20 in rottlerin-mediated tumor suppressive activity, we depleted Cdc20 by its shRNA in glioma cells. We found that down-regulation of Cdc20 inhibited cell growth in both glioma cells (Figure 6A). Moreover, depletion of Cdc20 enhanced cell growth inhibition induced by rottlerin in glioma cells (Figure 6A). We also observed that down-regulation of Cdc20 triggered cell apoptosis in glioma cells (Figure 6B). Cdc20 shRNA treatment led to more apoptotic cells induced by rottlerin compared with rottlerin alone or Cdc20 shRNA transfection alone in glioma cells (Figure 6B). Our invasion assay showed that depletion of Cdc20 suppressed glioma cell invasion (Figure 6C). Notably, Cdc20 shRNA enhanced cell invasion inhibition in rottlerin-treated glioma cells (Figure 6C). In line with this, depletion of Cdc20 retarded the cell migration in both U251 and SNB19 cells (Figure 7A). Furthermore, Cdc20 shRNA promoted inhibition of migration induced by rottlerin in glioma cells (Figure 7A). Our Western blotting analysis demonstrated that Cdc20 shRNA enhanced rottlerin-mediated inhibition of Cdc20 in glioma cells (Figure 7B). Consistently, Cdc20 shRNA promoted rottlerin-induced p21 level in both glioma cells (Figure 7B).

## DISCUSSION

Rottlerin increased HO-1 (heme oxygenase-1) expression via ROS (reactive oxygen species) pathway in human colon cancer cells [30]. Rottlerin was also exhibited its anti-angiogenesis function via inhibition of human microvascular endothelial cell proliferation and tube formation on Matrigel [31]. Moreover, rottlerin was reported to enhance IL-1β (interleukin-1β)-induced COX-2 (cyclooxygenase-2) expression via activation of p38 MAPK (mitogen-activated protein kinases) in breast cancer cells [32]. Lim et al found that rottlerin induced apoptosis via upregulation of NAG-1 through ERK (extracellular signal regulated kinase) and p38 MAPK pathway in colon cancer cells [32]. Furthermore, rottlerin inhibited PI3K (phosphoinositide 3-kinase)/Akt/mTOR (mammalian target of rapamycin) pathway, leading to autophagy and apoptosis in pancreatic cancer stem cells [33] and prostate cancer cells [34]. Lu et al. reported that rottlerin induced LRP6 (LDL-receptor related protein 6) degradation and inhibited both Wnt/β-catherin and mTORC1 signaling in prostate and breast cancer cells [35]. Recently, it has been reported that rottlerin inhibited pancreatic tumor growth in nude mice [36]. Notably, rottlerin suppressed cell growth via the dual inhibition of ERK and NF-κB and downregulation of Cyclin D1 in melanoma cells [37]. In the present study, we identified that rottlerin inhibited Cdc20 expression, resulting in inhibition of tumorigenesis in glioma.

Recently, Cdc20 was reported to be a key factor in maintaining tumorigenic glioma tumor initiating cells through degradation of p21 and regulation of Cdc25C (cell division cycle 25C), c-Myc, and Survivin [38]. In support of this concept, another study demonstrated that Cdc20 drives invasiveness and self-renewal in patient tumor-derived GSCs (glioblastoma stem-like cells) through pluripotenty related transcription factor SOX2 [39]. Moreover, using an orthotopic xenograft model overexpression of Cdc20 enhanced the GSCs to generate brain tumors, whereas depletion of Cdc20 retarded the GSCs to develop brain tumors [39]. Strikingly, it has been reported that Cdc20 induced the proliferation of primary glial progenitor cells [40]. Some studies showed that rottlerin inhibited cancer cell growth and induced apoptosis in a variety of human cancers [13, 14, 41, 42]. Moreover, rottlerin could suppress growth of human pancreatic tumors in nude mice [43, 44]. We also found that overexpression of Cdc20 enhanced glioma cell proliferation, whereas depletion of Cdc20 suppressed cell growth in glioma cells. Our results further showed that Cdc20 overexpression enhanced cell migration and invasion in glioma cells, while downregulation of Cdc20 retarded cell motility. Altogether, Cdc20 plays a pivotal role in regulation of cell growth, migration and invasion in glioma cells.

Since Cdc20 is identified as an oncoprotein in tumorigenesis, inactivation of Cdc20 could be useful for the treatment of human cancers. To this end, several Cdc20 inhibitors have been discovered. For example, TAME (tosyl-L-arginine methyl ester) reduced Cdc20 association with the APC and subsequent inhibited APC E3 ligase activity [45]. Moreover, pro-TAME with cell permeable activity disrupted the APC-Cdc20/Cdh1 interaction to reduce APC activation, leading to cell death in multiple cancer cell lines [45]. Notably, apcin (APC inhibitor) binds Cdc20 and prevents substrate recognition and competitively suppresses the ubiquitination of Cdc20 substrates [46]. Strikingly, apcin and pro-TAME synergized to enhance the mitotic fraction in human cancer cell lines [46]. In addition, compound 331 was found to be a potential drug selectively targeting glioma cells through down-regulation of Cdc20 [47]. NAHA, a N-alkylated amino acid-derived sulfonamide hydroxamate, inhibited the expression of Cdc20 in breast cancer cells [48]. Several natural agents were identified to inhibit the Cdc20 expression in human cancer cells. withaferin A exerted its anti-cancer activity through enhanced degradation of Cdc20 and Mad2 [49]. Ganodermanontriol (GDNT), a ganoderma alcohol from medicinal mushroom, inactivated Cdc20 in breast cancer cells [50]. Genistein, the predominant isoflavone in soybean enriched foods, was reported to exert its anti-carcinogenic properties through inhibition of multiple genes including Cdc20 in primary glioblastoma, rhabdomyosarcoma, hepatocellular carcinoma and human embryonic carcinoma cells [51]. Moreover, genistein controlled Cdc20 expression, leading to regulation of cell cycle in breast cancer cells [52]. Here, we identified that rottlerin could be a new Cdc20 inhibitor in glioma cells. Due to non-toxic nature, inactivation of Cdc20 by rottlerin could be a safer strategy for the treatment of glioma.

## MATERIALS AND METHODS

### Cell culture and experimental regents

Human glioma U251 cells and SNB19 cells were cultured in DMEM medium with 10% fetal bovine serum and 1% penicillin and streptomycin in a 5% CO2 at 37°C. Primary antibodies for Cdc20 (#14866, 1:1000) and p21 (#2947, 1:1000) were purchased from Abcam and Cell Signaling Technology, respectively. All secondary antibodies were purchased from Thermo Scientific. Lipofectamine 2000 was purchased from Invitrogen. Monoclonal anti-β-actin (A2228, 1:5000), rottlerin (CAS number 82-08-6, 85%), and MTT (3-4,5-dimethyl-2-thiazolyl-2, 5-diphenyl-2-H-tetrazolium bromide) were obtained from Sigma-Aldrich (St.Louis, MO).

### MTT assay

Cells were seeded in 96-well plate at a density of 5 × 10^3^ cells/well in a volume of 100 μl per well for 24 h and treated with different concentrations of rottlerin. After 48 h and 72 h, 10 μl MTT solution (0.5 mg/ml) was added to each well, and the plates were incubated for 4 h at 37°C with 5% CO_2_. Then, the liquid supernatant was removed and 100 μl DMSO was added in each well. The absorption was measured at 490 nm on the Multimode Reader of SpectraMax M5 (Moleucular Devices, US).

### Cell apoptosis assay

Cells were seeded at a density of 2 × 10^5^ cells/well in 6-well plate with different concentration of rottlerin. After 48 h, cells were harvested and washed with PBS, cells were suspended with 500 μl binding buffer with 5 μl FITC-conjugated anti-Annexin V antibody and 5 μl PI (Propidium iodide), and then analyzed with a FACScalibur flow cytometer (BD, USA).

### Cell cycle analysis

Cells were cultured in six-well plate at a density of 2 × 10^5^ cells/well overnight and treated with various concentration of rottlerin. After 48 h, cells were harvested and washed with PBS, and suspended with 70% cold alcohol for overnight in 4°C icebox. Then, cells were washed and suspended with 500 μl PBS. Then incubated with 100 μg/ml RNase and 50 mg/ml PI (Propidium iodide) at 37°C and then analyzed the cell cycle with a FACScalibur flow cytometer (BD, USA).

### Cell scratch assay

Cells were seeded in 6-well plate for overnight and reached almost 100% confluence. Then scratch the cells with a sterile 20 μl pipette tip. Wash the detached cells with PBS and incubate with different concentrations of rottlerin. The scratch was photographed with Box-Type Fluorescence Imaging Device (FSX100, OLYMPUS) at 0 h and 20 h.

### Cell invasion assay

Cell invasion assay was used to determine the invasive activity of glioma cells treated with rottlerin or Cdc20 cDNA or shRNA. Cells tranfected with Cdc20 cDNA or shRNA incubated with rottlerin or not with 200 μl serum-free medium in the up camber with Matrigel. Then added 500 μl DMEM medium with 10% FBS. The cells were incubated for 20 h at 37°C with 5% CO_2_ and then strained with Giemsa and photographed with Box-Type Fluorescence Imaging Device (FSX100, OLYMPUS).

### Quantitative real-time reverse transcription-PCR analysis

The total RNA was extracted with Trizol (Invitrogen, Carlsbad, CA) and reversed-transcribed into cDNA by RevertAid First Strand cDNA Synthesis Kit. PCR were performed using Power SYBR Green PCR Master Mix and the results were calculated by 2-⊿⊿Ct method. The primers used in the PCR reaction are: Cdc20, forward primer (5′- GAC CAC TCC TAG CAA ACC TGG -3′) and reverse primer (5′-GGG CGT CTG GCT GTT TTC A-3′); GAPDH, forward primer (5′- ACC CAG AAG ACT GTG GAT GG -3′) and reverse primer (5′- CAG TGA GCT TCC CGT TCA G- 3′).

### Western blotting analysis

The harvested cells were washed by PBS and lysed with protein lysis buffer. The concentrations of the proteins were tested by BCA Protein Assay kit (Thermo Scientific, MA). Same amount of protein samples were separated by electrophoresis in Sodium Dodecyl Sulfonate (SDS)-polyacrylamide gel and then transferred onto a Polyvinylidene Fluoride (PVDF) membrane, and then incubated with primary antibody at 4°C overnight. After washed with TBST for three times and incubated with second antibody at room temperature for one hour. Then the expression of protein was detected by electrochemiluminescence (ECL) assay [53].

### Transfection

Cells were seeded into 6-well plates and transfected with Cdc20 cDNA or Cdc20 shRNA or empty vector using lipofectamine 2000 following the instruction's protocol. After the transfection, the cells were subjected to further analysis as described under the results sections.

### Statistical analysis

All statistical analyses were conducted using GraphPad Prism 4.0 (Graph Pad Software, La Jolla, CA). Student's *t-test* was performed to evaluate statistical significance. *P* < 0.05 was considered as statistically significant.
